# Sleep Duration and Sleep Quality following Acute Mild Traumatic Brain Injury: A Propensity Score Analysis

**DOI:** 10.1155/2015/378726

**Published:** 2015-03-17

**Authors:** Ting-Yun Huang, Hon-Ping Ma, Shin-Han Tsai, Yung-Hsiao Chiang, Chaur-Jong Hu, Juchi Ou

**Affiliations:** ^1^Department of Emergency Medicine, Shuang-Ho Hospital, Taipei Medical University, New Taipei City 235, Taiwan; ^2^Department of Emergency Medicine, Taipei Medical University, Taipei 110, Taiwan; ^3^College of Public Health and Nutrition, Taipei Medical University, Taipei 110, Taiwan; ^4^Department of Neurosurgery, Taipei Medical University, Taipei 110, Taiwan; ^5^Translational Research Laboratory, Cancer Center, Taipei Medical University, Taipei 110, Taiwan; ^6^Department of Surgery, College of Medicine, Taipei Medical University, Taipei 110, Taiwan; ^7^Department of Neurology, Shuang-Ho Hospital, Taipei Medical University, New Taipei City 235, Taiwan; ^8^Department of Emergency Medicine, Shuang-Ho Hospital, Taipei Medical University, No. 291 Zhongzheng Road, Zhonghe District, New Taipei City 235, Taiwan

## Abstract

*Introduction*. Mild traumatic brain injury (mTBI) has been widely studied and the effects of injury can be long term or even lifelong. This research aims to characterize the sleep problems of patients following acute mTBI. *Methods*. A total of 171 patients with mTBI within one month and 145 non-mTBI controls were recruited in this study. The questionnaire, Pittsburgh Sleep Quality Index (PSQI), was used to evaluate seven aspects of sleep problems. A propensity score method was used to generate a quasirandomized design to account for the background information, including gender, age, Beck's Anxiety Index, Beck's Depression Index, and Epworth Sleepiness Scale. The effect was evaluated via cumulative logit regression including propensity scores as a covariate. *Results*. Before adjustment, about 60% mTBI patients and over three quarters of control subjects had mild sleep disturbance while one third mTBI patients had moderate sleep disturbance. After adjusting by the propensity scores, the scores of sleep quality and duration were significant between mTBI and control groups. *Conclusion*. Our study supports that sleep problem is common in mTBI group. After adjusting the confounders by propensity score, sleep duration and subjective sleep quality are the most frequently reported problems in mTBI patients within one month after the injury.

## 1. Introduction

More than a million people in the United States are affected by traumatic brain injury (TBI) annually [1]. The severe TBI typically results in disability or death, and TBI of any severity usually can affect the patient's physical, cognitive, and emotional wellbeing [2]. More than 80% of patients with TBI are classified as mild cases (mTBI), and most of mTBI patients may not have strongly and immediately uncomfortable feeling to this kind of injury. However, the effects of the mTBI can be on the long term or even lifelong [3–5]. Sleep problems are one category of the most commonly reported symptoms [6, 7]. Sleep disturbance is also associated with increased risk of depression and anxiety, which are common after an event with mental or physical stress [8]. However, the sleep problems that might occur following an mTBI have yet to be fully characterized.

Pittsburgh Sleep Quality Index (PSQI), which is divided into 7 components, is a questionnaire frequently used for the evaluation of sleep problems in clinical and healthy populations [9]. In previous studies, a 3-factor model of the PSQI provided more accurate results on sleep disturbances than a global analysis did [10–13]. In an observational study, one main problem is that the case (exposed) and control groups may not be comparable and the outcomes might not represent a causal effect. One solution is the propensity score introduced by Rosenbaum and Rubin [14] in order to control the distributions of the unbalance covariables between case and control groups.

Therefore, this study aimed to determine the patterns of sleep problem associated with the mTBI by use of the PSQI. Specifically, we performed an analysis by the propensity score model to describe the characteristics of sleep problems among the patients following acute mTBI.

## 2. Methods

### 2.1. Participants and Procedure

All of the mTBI patients aged ≥17 years who were admitted to any of the 3 affiliated hospitals of Taipei Medical University (TMU) between March 2010 and February 2013 were recruited. The definition of mTBI was based on the diagnostic criteria established by the American Congress of Rehabilitation Medicine, which consist of a Glasgow Coma Scale (GCS) score of 13–15 at presentation, loss of consciousness for <30 min, and normal head computed tomography findings. Patients who had a history of cerebrovascular disease, psychiatric comorbidities, epilepsy, alcohol abuse, sleep-wake modifying treatment, previous TBI, or severe systemic medical illness were excluded. In addition, volunteers who were older than 17 years old and did have no brain injury were recruited into the control group. The exclusion criteria for the control participants were the same as those for the mTBI patients. All patients were initially contacted by telephone and 675 mTBI patients were recruited. Among the mTBI patients, 171 (25.33%) provided informed consent and completed a baseline assessment during an initial evaluation within 1 month of experiencing an mTBI. The study protocol was approved by the Joint Institution Review Board at TMU.

### 2.2. Measures

*Pittsburg Sleep Quality Index (PSQI).* The PSQI is a 19-item self-reported instrument designed to measure a person's sleep quality and patterns of sleep. It contains 7 domains: duration of sleep, sleep syndrome, sleep latency, daytime dysfunction caused by sleepiness, sleep efficiency, overall sleep quality, and use of sleep medications [9]. Each domain is scored from 0 to 3, with a higher value indicating poorer sleep quality and a clinical cutoff point of 5. The Chinese version of the PSQI has been validated [15]. 

*Epworth Sleepiness Scale (ESS).* Daytime sleepiness was evaluated using the validated Chinese version of the ESS. Each question is rated on a 4-point scale (0, never; 3, highly likely) [16], with a clinical cutoff point of 9. The Cronbach's alpha of the Chinese ESS is 0.81 and the test-retest reliability is 0.74. 

*Beck Depression Inventory (BDI) II.* The BDI is designed to measure depressive symptoms. This study used the Chinese version of the BDI II [17]. This questionnaire contains 21 items, scored on a scale of 0 (no problem) to 3 (severe problems). The total possible score ranges from 0 to 63, with a clinical cutoff point of 9. A higher BDI score indicates greater severity of depression [18].

*Beck Anxiety Inventory (BAI).* The Chinese version of the BAI was used. The BAI is a 21-item self-reported questionnaire designed to assess the symptoms of anxiety [19]. Each item is rated on a 4-point scale. The total possible score ranges from 0 to 63, with a clinical cutoff point of 7. A higher score indicates more severe anxiety [20].

### 2.3. Statistical Analysis

The number of participants for each PSQI component was calculated, and differences in trends between the mTBI and control groups were compared using the Cochran-Armitage test. Also, the association between scales and the other confounders was assessed via Spearman's correlations. In this study, the participants in the control group were assumed to represent the general population. The control participant recruited without matching the age and gender of the mTBI group. In order to generate a quasirandomized design, the propensity score method was used to account for selection biases and potential confounding factors. The propensity scores were calculated by the logistic regression to estimate the probability of each patient on the basis of age, sex, and questionnaires. The best model was selected according to AIC stepwise algorithm. The effects for each component were assessed via cumulative logit regression. In all statistical tests, a *P* value of *P* < 0.05 was considered significant and all tests were 2-tailed. The analyses were conducted using R software version 3.1.1.

## 3. Results

### 3.1. Demographic Information

In this study, we recruited 675 mTBI patients and 186 control participants, of whom 171 and 145 subjects, respectively, completed the PSQI and other questionnaires and signed the informed consent. The participants' demographic information is shown in Table 1. The percentages of men in two groups were not significantly different: 32% in the mTBI group and 38% in the control group (*P* = 0.06). The average years of education and percent of married participants were not different between the mTBI and control groups. The mean age of the mTBI patients was significantly higher than that of the control participants (38.57 y versus 32.18 y; *P* < 0.001). More than 50% of the mTBI patients suffered from headache problems, and their BAI, BDI, and global PSQI scores were higher than those of the control group. As shown in Table 1, more than half of the mTBI patients had sustained their brain injuries in a motor accident, and a third of the group had sustained their injuries during a fall.

### 3.2. Analysis of PSQI Components before Adjustment

**Figure 1:**
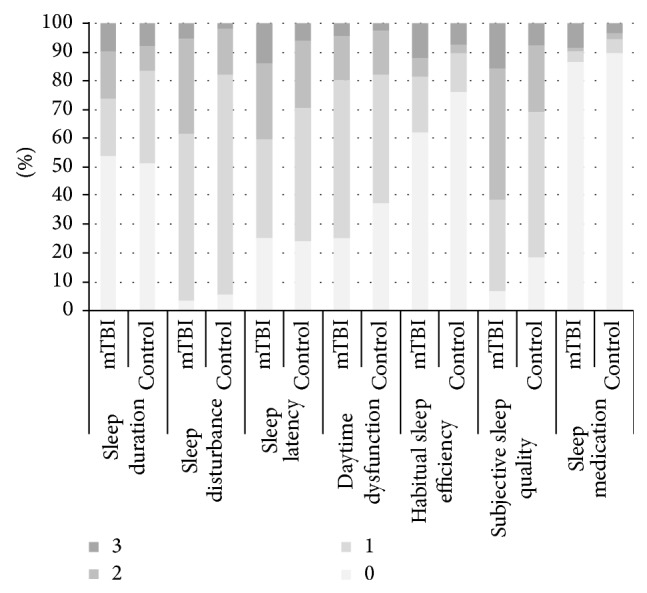
Percentage of each score for seven sleep components.

The stratified numbers of participants in each category of PSQI components are shown in Table 2 and the percentage of each score for each component are shown in Figure 1. The global PSQI score was different between two groups. However, four of them were not significantly different between mTBI and control groups. For both two groups, most of participants had no sleep duration problem, mild sleep latency, mild daytime dysfunction, and no use of sleep medication. The percentages of participants differed significantly between the mTBI and control groups in three components, sleep disturbance, habitual sleep efficiency, and subjective sleep quality. About 60% mTBI patients and over three quarters control subject had mild sleep disturbance while one third mTBI patients had moderate sleep disturbance. The percent of mild habitual sleep efficiency in mTBI group was significantly higher than that in the control group. Moreover, the percentage of moderate seep quality in mTBI group was higher than that in control groups. Overall, mTBIs showed more sleep disturbance, worse sleep efficiency and poorer sleep quality.

### 3.3. Associations

The *P* value of the associations between sleep components and the other confounders are shown in Table 3. The components, sleep duration in mTBI group, daytime dysfunction in control group, and sleep medication in both groups, were related to the confounder, age. The BAI/BDI scores were not associated with sleep duration and the use of sleep medication for control group. The ESS was related to three components, sleep latency, daytime dysfunction, and subjective sleep quality.

### 3.4. Analysis of PSQI Subscores after Adjustment

**Figure 2:**
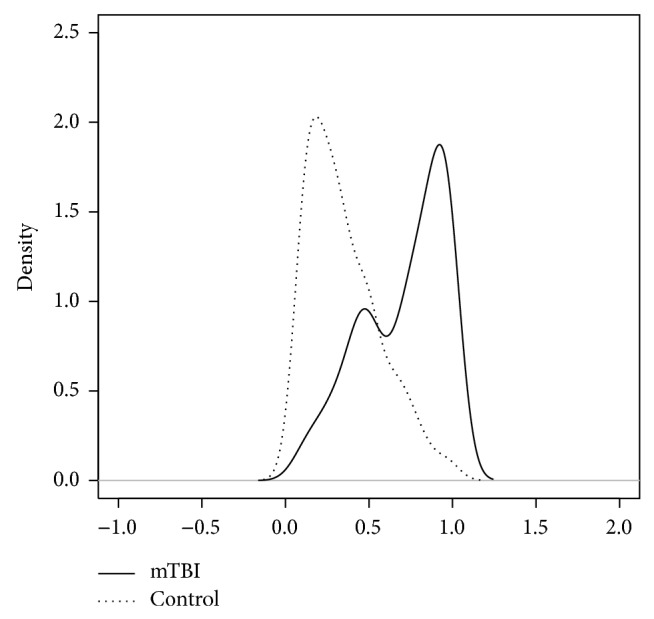
Propensity score density for mTBI (solid line) and control (dotted line) participants.

The confounders were assessed for both groups via the logistic regression. After stepwise, the final model included four covariates, age, sex, BAI, and headache (as shown in Table 4). The propensity scores were calculated according to the final model above and the density of the propensity scores for two groups is shown in Figure 2. The results of group effect with and without propensity scores are shown in Table 5. The crude results from the cumulative logit models without propensity scores showed the significance in three components, sleep disturbance, habitual sleep efficiency, and subjective sleep quality. This is the same result as the result of the trend test. However, the results were different after adjusting by propensity scores. The cumulative logit model results for two significant group effects are shown in Table 6. The proportional odds ratio of comparing mTBI to control groups on sleep duration was 0.49. For the mTBI group, the odds of sleep duration less than 5 hours versus the sleep duration more than 5 hours are 0.49 times lower than those for controls after adjusting by propensity scores. For the other outcome, subjective sleep quality, the odds of very poor sleep quality versus the combined the other three categories are 2.075 times greater than those for controls.

## 4. Discussion and Conclusion

All of the mTBI patients in this study had suffered nonblast injuries [21–23]; therefore, the results represent a nonwar-related scenario in the general population. Our results indicate that patients with mTBI experience increased risk of sleep problems in comparison with non-mTBI controls. After adjusting all other confounders, such as gender, age, depression, anxiety, and daytime sleepiness by propensity score analysis, the mTBI patients have different sleep duration and subjective sleep quality in the self-reported questionnaire. These findings are compatible with those from previous studies, which evaluated patients 3 months and 3 years after TBI [7, 24, 25]. Our study results also indicate that sleep problems typically occur in the acute stage immediately after an mTBI. Moreover, two sleep components, in terms of sleep duration and subjective sleep quality, were significantly different from the control group while the others showed no difference after adjusting by propensity scores as a covariate. In the study of Fichtenberg et al., poor sleep quality, including limited duration and efficiency of sleep, and insomnia were the 2 major sleep problems to affect TBI patients [26]. In two other previous studies, the quantitative electroencephalograms (qEEG) of mTBI and non-mTBI patients showed no significant differences [27, 28]. However, in the study of Khoury et al., sleep architecture differed significantly between mTBI with pain and control participants but sleep architectures of two groups were within normal ranges [29].

One finding of particular interest was that sleep problems following mild traumatic brain injury can be associated with anxiety and depression. Therefore, the group effect was evaluated after adjusting by the propensity score model including anxiety and depression scores in order to achieve the quasirandomized observation study. In the mTBI group, we observed that the PSQI subscore for sleep disturbance was moderately to strongly associate with the BAI score. We suggest that some features of anxiety, such as increased arousal at night, can lead to sleep disruption and then result in changes of sleep duration and subjective sleep quality [30].

There are a few limitations in the study. First, the subjective data were collected instead of objective data, such as adrenocorticotropic hormone (ACTH) and cortisol which were associated with sleep quality [31–33]. Second, long-term follow-up studies need to be considered for elucidating the disease course of sleep problems following mTBI.

In conclusion, our study results indicate the characteristics of sleep problems following mTBI. The patients with mTBI had significantly different sleep duration and sleep quality after adjusting all other confounders. These findings could potentially increase physicians' understanding of the consequences of the mTBI. Studies with longer-term followup and analyses of biomarkers such as ACTH and cortisol are recommended to facilitate the raising an optimal management of sleep disturbance in mTBI patients.

## Figures and Tables

**Table 1:** Demographic data of the mTBI and control groups.

Variables	mTBI	Control	*P* value
*N*	171	145	
Glasgow outcome score	14.98 (0.12)	15	0.99
Age at injury, mean (SD)	38.57 (15.09)	32.18 (10.63)	<0.001
Men, *n* (%)^#^	56 (32.74%)	38 (26.21%)	0.06
Years of education (SD)	15.33 (1.95)	14.93 (2.23)	0.88
Married, *n* (%)^#^	59 (34.50%)	44 (30.34%)	0.27
Employed, *n* (%)^#^	83 (48.53%)	70 (48.28%)	0.47
Headache, *n* (%)^#^	107 (62.57%)	30 (20.69%)	<0.001
Mechanism of injury, *n* (%)			
Transportation accident	92 (53.80)	—	
Fall	57 (33.33)	—	
Other	22 (12.87)	—	
Psychological evaluations			
BAI	9.30 (10.14)	2.62 (3.62)	<0.001
BDI	8.75 (8.13)	5.18 (7.24)	<0.001
ESS	7.33 (4.28)	6.49 (3.51)	0.12
PSQI	7.23 (3.92)	5.65 (3.45)	<0.001

^#^mTBI and control groups compared using a proportional test.

SD: standard deviation.

**Table 2:** Numbers (percentages) of participants with each PSQI subscore in the mTBI and control groups.

PSQI		Number (%)		*P* value
		mTBI *n* = 171	Control *n* = 145	
Sleep duration	>7 h	92 (54)	74 (51)	0.44
	6-7 h	34 (20)	47 (32)	
	5-6 h	28 (16)	12 (8)	
	<5 h	17 (10)	12 (8)	

Sleep disturbance	None	6 (4)	8 (6)	<0.01
	1–9	99 (58)	111 (77)	
	10–18	57 (33)	23 (16)	
	19–27	9 (5)	3 (2)	

Sleep latency	<15 min and not during the previous month	43 (25)	35 (24)	0.10
	16–30 min and less than once/wk	59 (35)	67 (46)	
	31–60 min and once or twice/wk	45 (26)	34 (23)	
	>60 min and >3 times/wk	24 (14)	9 (6)	

Daytime dysfunction	No problems	43 (25)	54 (37)	0.07
	Minor problems	94 (55)	65 (45)	
	Considerable problems	26 (15)	22 (15)	
	Major problems	8 (5)	4 (3)	

Habitual sleep efficiency	≥85%	106 (62)	110 (76)	0.01
	75%–84%	33 (19)	20 (14)	
	65%–74%	11 (6)	4 (3)	
	<65%	21 (12)	11 (8)	

Subjective sleep quality	Very good	11 (6)	27 (19)	<0.01
	Relatively good	55 (32)	73 (50)	
	Relatively poor	78 (46)	34 (23)	
	Very poor	27 (16)	11 (8)	

Use of sleep medication	Never during the previous month	148 (87)	130 (90)	0.14
	Less than once/wk	6 (4)	7 (5)	
	Once or twice/wk	2 (1)	3 (2)	
	≥3 times/wk	15 (9)	5 (3)	

**Table 3:** *P* value of Spearman's correlation among the PSQI subscores and age, BDI, BAI, and ESS score.

	Group	Age	BAI	BDI	ESS
Sleep duration	mTBI	0.01	<0.01	0.02	0.6
	Control	0.16	0.22	0.65	0.14

Sleep disturbances	mTBI	0.17	<0.01	<0.01	0.04
	Control	0.58	<0.01	0.01	0.04

Sleep latency	mTBI	0.67	<0.01	<0.01	0.55
	Control	0.15	<0.01	<0.01	0.16

Daytime dysfunction	mTBI	0.09	<0.01	<0.01	<0.01
	Control	0.02	<0.01	<0.01	<0.01

Habitual sleep efficiency	mTBI	0.29	0.01	<0.01	0.62
	Control	0.13	0.03	0.03	0.10

Subjective sleep quality	mTBI	0.92	<0.01	<0.01	0.06
	Control	0.89	<0.01	<0.01	<0.01

Use of sleep medication	mTBI	0.04	<0.01	0.01	0.44
	Control	0.04	0.16	0.15	0.73

**Table 4:** Results of propensity score model.

	Estimate	*P* value
Age	0.05	<0.01
Sex	−1.28	<0.01
BAI	0.19	<0.01
Headache	1.74	<0.01
BDI	−0.02	0.38
ESS	−0.02	0.58

After stepwise		
	Estimate	*P* value

Age	0.05	<0.01
Sex	−1.32	<0.01
BAI	0.17	<0.01
Headache	1.71	<0.01

**Table 5:** Results of mTBI effect via cumulative logit model with or without propensity score as a covariate.

Model	Estimate	sd	*z*-value	*P* value
Sleep duration				
Crude	0.0694	0.2136	0.3249	0.75
Propensity score	−0.7122	0.2685	−2.6525	<0.01
Sleep disturbance				
Crude	0.9691	0.2496	3.8827	<0.01
Propensity score	−0.0498	0.3087	−0.1613	0.87
Sleep latency				
Crude	0.2998	0.2496	3.8827	0.14
Propensity score	−0.3154	0.2477	−1.2734	0.20
Daytime dysfunction				
Crude	0.4126	0.2153	1.9166	0.06
Propensity score	−0.1134	0.2617	−0.4332	0.66
Habitual sleep efficiency				
Crude	0.6535	0.2459	2.6572	<0.01
Propensity score	0.0722	0.3016	0.2394	0.81
Subjective sleep quality				
Crude	1.1907	0.2201	5.4096	<0.01
Propensity score	0.7300	0.2532	2.8831	<0.01
Use of sleep medication				
Crude	0.3366	0.3522	0.9557	0.34
Propensity score	−0.5840	0.4656	−1.2544	0.21

**Table 6:** Results of the proportional-odds cumulative logit model.

Model		Estimate	sd	95% CI	Odds ratio
Sleep duration					
Intercept	log(*P*(>7 h)/(1 − *P*(>7 h)))	1.2207	0.2641	(0.70, 1.74)	
Intercept	log(*P*(>6 h)/(1 − *P*(>6 h)))	2.4835	0.2926	(1.91, 3.06)	
Intercept	(*P*(>5 h)/(1 − *P*(>5 h)))	3.5940	0.3403	(2.93, 4.26)	
mTBI		−0.7122	0.2685	(−1.24, −0.19)	0.491

Subjective sleep quality					
	log(*P*(0)/(1 − *P*(0)))	−0.8032	−0.2837	(−1.32, −0.28)	
	log(*P*(≤1)/*P*(>1))	1.1130	2.1525	(1.11, 2.15)	
	log(*P*(≤2)/*P*(>2))	3.2958	4.6749	(3.30, 4.67)	
	0: very good; 1: relatively good; 2: relative poor; 3: poor				
mTBI		0.7300	0.2532	(0.23, 1.23)	2.075
